# Placebo Effects on the Enjoyment of Physical Activity and Performance among Kindergarten Children: A Randomized Controlled Trial

**DOI:** 10.3390/ejihpe14080161

**Published:** 2024-08-22

**Authors:** Marlies Stopper, Albert Wabnegger, Anne Schienle

**Affiliations:** Clinical Psychology, University of Graz, 8010 Graz, Austria

**Keywords:** placebos, open-label placebos, children, kindergarten, enjoyment, physical activity, performance

## Abstract

Studies with adults and school children have shown that placebos can enhance motivation and performance in physical activities. This study aimed to investigate whether similar effects are present in kindergarten-aged children. A total of 101 children (58 girls, 43 boys) aged 3 to 6 years were randomly assigned to one of two groups that either received a deceptive placebo (DP: “magic potion”) or a nondeceptive placebo (NDP: “water”) to enhance physical abilities. Each child completed three tasks (sprinting; balancing: standing on a balance board; strength: using a handheld dynamometer) both with and without the placebo. The variables assessed included task performance, enjoyment, and expected and perceived placebo efficacy (measured with nonverbal pictorial rating scales). Results showed that both the DP and NDP increased speed. For strength, balance, and task enjoyment (which was very high), no placebo-induced changes were observed. Expected efficacy was higher for the DP; perceived efficacy did not differ between DP and NDP. In conclusion, reported outcome expectations indicated that kindergarten-aged children were already able to differentiate between the two types of placebos which exhibited positive effects concerning running performance. This encourages further research on using nondeceptive placebos to enhance physical activity, which is crucial for children’s overall health.

## 1. Introduction

Physical activity plays a crucial role in the promotion and maintenance of physical and mental health [1]. This not only applies to adults but also to children for whom exercise features a particularly important aspect in their motor, cognitive, and socio-emotional development [2]. Despite the known benefits of regular exercise, the majority of children and adolescents do not engage in sufficient physical activity according to the recommendations of the World Health Organization [3]. This is concerning as insufficient physical activity is associated with an increased risk of becoming overweight and developing obesity and associated somatic and mental health conditions [4,5]. To prevent negative health effects, it is essential to identify simple, effective interventions that promote physical activity among children and boost their motivation for sports.

Numerous studies with adults [6,7] have indicated that prescribing placebos (a sham treatment in the form of an inert substance or procedure) can be one way to effectively enhance sports motivation and performance. For instance, Ross and colleagues [8] found that a saline injection, labeled as a medication to stimulate red blood cell production, improved endurance runners’ performance. Similar effects have been observed in muscle strength; Kalasountas et al. [9] demonstrated that a placebo presented as a potent amino acid combination with immediate strength benefits significantly enhanced college students’ performance in bench and leg press tasks. Villa-Sánchez et al. [10] discovered placebo-induced improvements in balancing through a single-legged balance test, where participants believed they had received electrical nerve stimulation to enhance postural control. In a more recent study, the same research group [11] found that a placebo group receiving sham electrical stimulation showed better reaction times and reported less mental and physical fatigue. These findings indicate that placebos can influence both objective and subjective performance measures.

In contrast, the body of research for children is limited, comprising only four studies that specifically addressed the influence of placebos on physical fitness [12,13,14,15]. In one study, Fanti-Oren and colleagues [12] found that normal-weight children aged 6 to 13 years demonstrated increased endurance in a treadmill test after having received a placebo being labeled as a drink that “strengthens muscles and increases energy”. In two other studies conducted by the same authors, similar effects were observed in overweight children aged between 8 and 13 years [13,14]. Finally, Szabo [15] identified positive effects of a placebo (“performance enhancer”) on the sprinting speed of kayak athletes (aged 10–16 years). Due to a lack of research, it remains unclear whether equivalent findings on deceptive placebos (DPs) can be assumed in younger children.

Aside from that, there is little research on the influence of nondeceptive placebos (NDPs: honestly administered placebos) in children. In contrast to DPs, where the placebo recipient is not informed about the true nature of the treatment, NDPs have become a favored approach due to the consideration of ethical aspects such as informed consent and autonomy [16]. However, all studies examining the influence of placebos on physical performance in children did so by the administration of a deceptive placebo (as reviewed by [17]).

The current study aimed to examine the influence of both deceptive and nondeceptive placebos on the enjoyment of physical activity and performance in children aged 3 to 6 years. To gain more insight into the influence of placebos on different forms of physical activity, the children completed three tasks: sprinting, balancing, and using a handheld dynamometer. The children received the placebo in the form of a spray (water) for oral administration. In the deceptive placebo condition, they were informed that they would receive a “magic potion” that was going to enhance their physical abilities. In the NDP condition, each child filled the spray bottle with water together with the experimenter and was told that the water would improve their physical abilities. Participants either received the DP or the NDP and were tested twice (with vs. without the placebo) each serving as their own control. Moreover, we assessed expected and perceived treatment efficacy. We hypothesized that following the administration of the placebo (DP and NDP), children would report heightened enjoyment in the tasks and demonstrate enhanced performance, reflected in increased speed, muscle force, and balance control.

## 2. Materials and Methods

### 2.1. Participants

**Figure 1:**
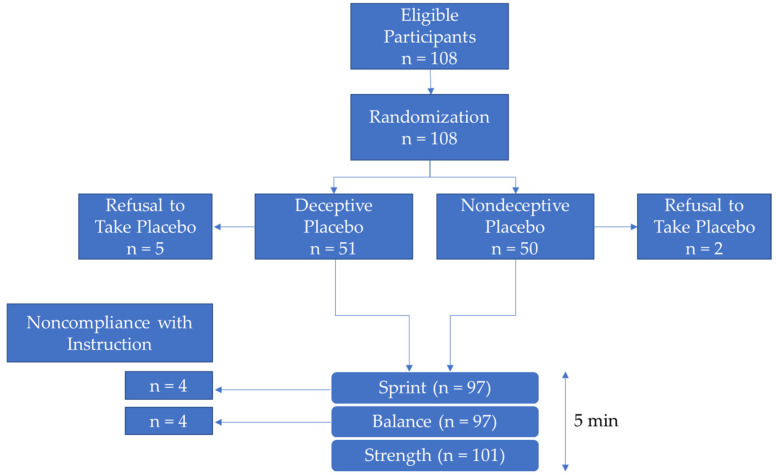
Flow chart.

A total of 108 children were enrolled in the study. Seven children refused to take the placebo, leaving 101 subjects in the final sample (see flow chart in Figure 1). Placebo refusal rates did not differ between the two groups (Z(1) = 1.15, *p* = 0.44).

Fifty-one children were randomized to the deceptive placebo group (DP) and fifty children to the nondeceptive placebo group (NDP; see Table 1 for group characteristics). The two groups did not differ in their gender ratio (χ^2^(1) = 0.01, *p* = 0.91), body mass index (t(99) = 0.66, *p* = 0.51), and mean age (t(99) = 0.39, *p* = 0.70).

The inclusion criteria required participants to be between 3 and 6 years old. Exclusion criteria were reported diagnoses (by parents or kindergarten teachers) of mental/physical disorders, and poor German language skills.

The sample size had been determined via G*Power (Version 3.1.9.7) based on effect sizes reported in previous placebo research with children [18]. For a medium effect size of f = 0.25, with an alpha error probability of 0.05 and a power of 0.95, a sample size of 36 subjects was required. All parents/children interested in participating in the study were tested, resulting in a larger sample size than originally intended.

The study followed the Declaration of Helsinki and received approval by the ethics committee of the University of Graz (GZ. 39/62/63 ex 2023/24) and the Styrian Government Department 6 of Education and Society (Pedagogical Quality Development). The study was preregistered on the German Register for Clinical Studies (DRKS00033408).

### 2.2. Design

In this randomized trial, participants either received the deceptive placebo (DP) or the nondeceptive placebo (NDP) and were tested twice (with vs. without the placebo).

**Figure 2:**
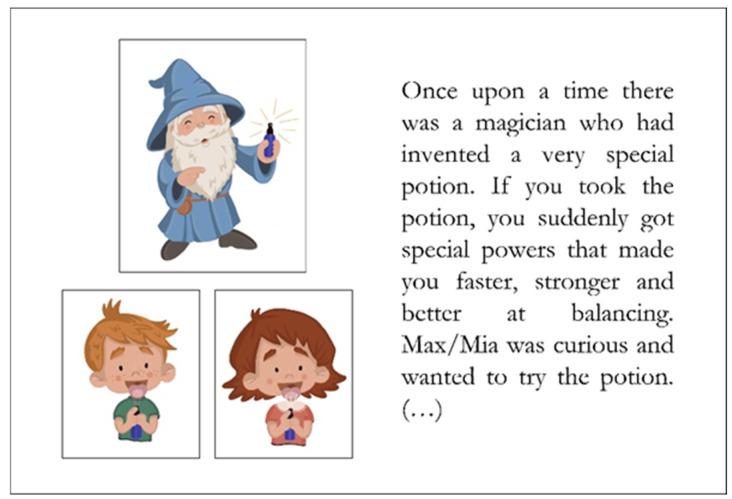
Cover story for the deceptive placebo (illustrations by M. Stopper).

Placebo: The placebo was tap water filled into a 30 mL blue glass bottle with a spray head. For the DP, the water was already in the bottle, whereas the water for the NDP was poured into the bottle in front of the participants while emphasizing that it was regular water. Subjects were then instructed to take three pump doses orally. The DP was introduced as a “magic potion”. For this purpose, subjects were given a short comic (Figure 2) that was read to them by the experimenter and told the story about a child who develops “special powers” after taking a magic potion brewed by a magician. Male subjects were given a male comic version starring a boy called “Max”, while female subjects were given a female comic version starring a girl called “Mia”.

Physical activities and measures: The children completed three activities (speed, balance, strength).

Sprinting speed: A stopwatch was used to measure the time (in seconds) it took for the children to cover a distance of 8 m.

Balance control: The children were asked to stand with their feet hip-width apart on a balancing board (MFT Challenge-Disc 2.0/USB) for 20 s. The board consisted of a flat round surface mounted on a fulcrum, allowing it to tilt in various directions. Corresponding software (Bodyteamwork, 2.0) installed on a tablet was used for giving visual feedback on one’s ability to hold balance through the activation of different muscle groups. This happened in the form of a green dot that was displayed on the screen and moved according to the tilting movements being executed on the board. Subjects were instructed to distribute their weight in a way so that the dot remained in the center of the screen, which reflected good balance control. The score varied from 1 to 5 with smaller numbers indicating better balance control.

Muscle force: Handgrip strength was measured using a calibrated handheld dynamometer (Gripx EH101YL), which is adjustable to accommodate different hand sizes. The participants were instructed to stand comfortably with their feet hip-width apart while holding the dynamometer with their dominant hand at a 90 degree angle. Subsequently, they were told to grip the dynamometer handle for 5 s as forcefully as possible without other body movements. The maximum force exerted during each condition was measured in kilograms.

### 2.3. Procedure

The study was conducted from February to May 2024 in six kindergartens (in the sports rooms). Contact was first made with the children’s parents/caregivers who received a flyer, a study information sheet (See Supplementary Material S1), and a consent form via e-mail. Those who consented to their child’s participation were asked to sign the form and return it to the kindergarten management, who submitted all signed consent forms to the experimenter prior to the start of the study.

On the day of the testing, children who were allowed to participate were checked for exclusion criteria. Moreover, participants’ age, weight, and height (body mass index: BMI) were assessed. Participants then completed the physical activities (in randomized sequence) twice under identical conditions, once with, and once without the placebo (randomized sequence). In the DP condition, children were instructed: “If you spray the magic potion on your tongue, you will suddenly become much faster, stronger, and better at balancing”, while children in the NDP condition received the same instruction with the exception that the word “magic potion” was replaced by the word “water”.

### 2.4. Rating Scales

Participants were provided with three nonverbal pictorial scales to evaluate the expected and perceived efficacy of the placebo as well as their enjoyment of the motor activities. Before the three tasks (sprint, balance, strength), they were asked how much they believed that the placebo would help them with the task performance: thumbs-up (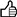, scored as 3), thumbs horizontal (scored as 2) or thumbs-down (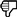, scored as 1). Perceived efficacy was rated with the same rating scale.

**Figure 3:**
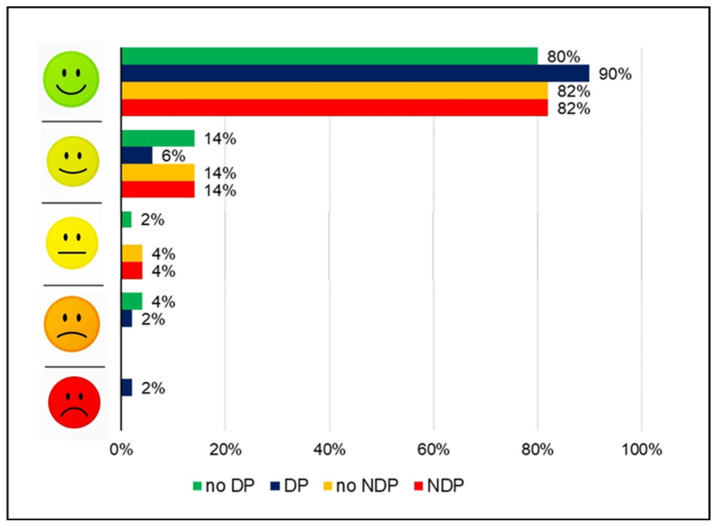
Enjoyment (DP: deceptive placebo; NPD: nondeceptive placebo).

After completing all three tasks (with/without placebo), the children were asked to rate their enjoyment of the activities on a 5-point scale with facial expressions ranging from a broad smile (happy, scored as 5) to a deep frown (unhappy, scored as 1); see Figure 3.

### 2.5. Statistical Analysis

Associations between PLACEBO TYPE (DP, NDP), INTAKE (with/without placebo), SEX (female, male), and the dependent variables (speed, balance, strength) were analyzed with three separate linear mixed models. Body mass index (BMI) was inserted as a covariate (mean-centered) in all analyses because height and weight influence physical performance. Moreover, since children were tested in six different locations (kindergartens), this was also considered in the analyses.

First, a null model was built, with a random intercept for KINDERGARTEN and SUBJECT (children nested in kindergartens). Next, the factors PLACEBO TYPE, INTAKE, and SEX were added to the model. Thus, the final model was as follows: DV (speed, balance, strength) ~ 1 + Intake + Placebo_Type + Sex + BMI + Intake: Placebo_Type + Intake: Sex + Placebo_Type: Sex + Intake: Placebo_Type: Sex + (1|subject) + (1|Kindergarten).

Frequencies (e.g., for outcome expectancy) were compared via Chi-squared tests.

For single tasks, children were excluded from the analyses when they did not follow the instructions (speed: *n* = 4 children did not run or stopped running in the middle of the task; balance: *n* = 4 children stepped off the board during the task). Computations were conducted with SPSS (version 29) and Jamovi 2.3.28.

## 3. Results

Descriptive statistics for the performance measures are displayed in Table 2.

Speed: The main effects INTAKE as well as SEX were statistically significant. Children ran faster with placebos (M: 3.07, CI [2.75–3.39], SE: 0.13) than without placebos (M: 3.18, CI [2.86–3.50], SE: 0.13). Boys (M: 3.05, CI [2.73–3.37], SE: 0.13) were faster than girls (M: 3.21, CI [2.89–3.53], SE: 0.13). All other effects were statistically non-significant (all ps > 0.128; Table 3).

Balance: The interaction between PLACEBO TYPE and INTAKE reached statistical significance (Table 4). Without the placebo, the NDP group performed better (M: 3.25 CI [2.84–3.66], SE: 0.19) than the DP group (M: 3.70 CI [3.29–4.11], SE: 0.19). In the condition with placebo administration, there was no difference between NDP (M: 3.35 CI [2.94–3.76], SE: 0.19) and DP (M: 3.44 CI [3.02–3.85], SE: 0.19).

Strength: For strength, no statistically significant effects were observed (all ps > 0.58; Supplementary Table S1).

Enjoyment: Reported enjoyment of the physical activities was very high and did not differ between the placebo and no-placebo condition: 86% of the children selected the most happy-looking face when under the placebo compared to 81% in the no-placebo condition (Figure 2).

Expected Efficacy: Five participants did not provide a rating (and stated that they “did not know” how effective the placebo would be); the remaining sample of *n* = 96 was analyzed. Two subjects—one in the DP group, the other in the NDP group—reported the placebo to be “not effective” (*n* of cell = 1), which is why the two categories “not effective” and “moderately effective” were merged into one category (*n* = 10). More participants in the DP group (96%) than in the NDP group (84%) expected the placebo to be “effective”, χ^2^(1) = 4.02, *p* = 0.045, φ = 0.21).

Perceived Efficacy: Both types of placebos were perceived as equally effective (DP: 94%; NDP: 94%).

## 4. Discussion

The current study was the first to examine the effects of deceptive and nondeceptive placebos on the enjoyment of physical activities and performance measures in kindergarten-aged children.

The placebo (both DP and NDP) increased running performance, likely increasing effort and engagement in the task. Similar results have been shown in a study on kayak athletes (10–16 years old), where a deceptive placebo increased heart rate and speed during a 2 min sprint [15]. Moreover, the anticipatory heart rate before the sprint was higher in the placebo group, pointing to positive expectancy effects. When individuals believe they are receiving a performance-enhancing treatment, it can boost their motivation. Placebo-induced enhancement of motivation is well documented in placebo research. For example, college students who were told they received a cognitive enhancer (actually a placebo) showed better performance in memory and concentration tasks [19]. Their belief in the enhancement boosted their motivation to perform well, leading to higher test scores. Moreover, students completed more exercises of relaxation training and patients with chronic pain completed more physical therapy sessions [20,21,22]. Thus, placebos can motivate individuals to engage in specific activities. This is further supported by the goal-activation model of the placebo effect, which suggests that the placebo response is enhanced when the motivation and expectation to experience a particular placebo response are aligned [23].

In contrast, no placebo-related effects were observed for balance and strength, which differs from previous studies with adults using similar tasks. For instance, Villa-Sánchez and colleagues [10] found that a placebo (a sham electrical device) improved participants’ stability while standing on their dominant foot. Additionally, the placebo enhanced their subjective perception of stability. Other studies have reported placebo-induced performance enhancements in tasks like bench press, leg press, hand press, and weight lifting [7]. The differing findings in this study may be related to the children’s lower familiarity with these physical tasks. None of them had used a handheld dynamometer before, and only one child had tried a balance board. Additionally, the results showed that balancing performance differed between the DP and NDP even without placebo administration, indicating baseline differences in performance.

Moreover, while the placebo likely enhanced task engagement, this does not necessarily translate to improved performance. In the case of the handheld dynamometer, performance gains are linked to muscle strength, whereas on the balance board, fine motor skills are crucial for success. These factors—pure force and motor skills—are largely independent of placebo-induced motivation. Therefore, motor tasks that involve resistance (e.g., endurance runs) might be more effective in eliciting placebo responses.

Overall, the children enjoyed the tasks (sprint, balance, strength) greatly, with 86% choosing the facial expression with a broad smile to describe their experience. While the children did not differentiate between the placebo types regarding enjoyment, their expectations concerning the treatment outcome differed, with higher ratings for the “magic potion” (deceptive placebo). This finding aligns with reports from adults indicating that deceptive placebos are perceived as more effective compared to nondeceptive placebos [24,25,26]. The present findings demonstrate that kindergarten-aged children were already able to differentiate between the two types of placebos. The clear instruction and demonstration, such as filling the water for the nondeceptive placebo (NDP) into the bottle in front of the participants, helped the children understand the concept.

Ratings for the perceived efficacy of the DP and NDP did not differ after the treatment, with almost all children (94%) reporting positive placebo effects. In adult samples, heterogeneous findings have been observed when directly comparing the outcomes of DP and NDP treatment. Some studies found no differences in efficacy (e.g., [27]), while others reported better effects for DPs compared to NDPs (e.g., [28]).

This study has several limitations. First, we studied a self-selected sample, so the results cannot be generalized to other groups of kindergarten children. However, the large sample size is a clear asset in this investigation.

Second, for ratings of task enjoyment, we observed ceiling effects, likely preventing placebo-induced changes.

Third, future studies should focus on endurance-orientated tasks to better capture placebo-related motivation effects on sports performance (e.g., biking, swimming, ball games). Moreover, investigations should test the effects of repeated placebo administration [17]. In the present study, a single-dose placebo was provided that induced short-term effects. However, to be practically relevant, the long-term effects of placebo treatment need to be demonstrated. Regular administration of placebos could encourage children to develop a lasting habit of engaging in physical activity.

Fourth, a small percentage of children (6%) refused to take the placebo, with some mentioning they were not allowed to take things from strangers. These comments underline the importance of the social component of placebo administration. Typically, children receive placebos from parents, caregivers, or other trusted individuals (e.g., trainers; see [15]). It would be highly interesting to explore whether similar or different effects are observed when the placebo provider is familiar with the recipient.

## 5. Conclusions

This study demonstrated that placebo treatment can enhance sprint performance in kindergarten children. Both deceptive and nondeceptive placebos were equally effective. Given that nondeceptive placebos avoid ethical concerns, they should be preferred. In the future, it seems promising to test the effects of a nondeceptive placebo on the enjoyment and performance in endurance sports or the regular engagement in athletic activities as placebo-related motivation effects can be expected in these domains. Since developing a habit of physical activity in childhood often leads to continued activity in adulthood, the use of placebos could play a small but meaningful role in promoting healthier lifestyles.

## Figures and Tables

**Table 1:** Group characteristics.

	Deceptive Placebo Group *n* = 51	Nondeceptive Placebo Group *n* = 50
Sex (male/female)	22/29	21/29
Age (months)	60.4 ± 9.11	61.1 ± 10.1
Weight (kg)	19.9 ± 3.45	19.2 ± 2.87
Height (cm)	112 ± 7.05	111 ± 6.86
BMI (kg/m^2^)	15.8 ± 1.58	15.6 ± 1.50

**Table 2:** Performance (means, ±standard deviations) in the two groups with vs. without placebo administration.

	Deceptive Placebo Group		Nondeceptive Placebo Group	
	with	without	with	without
	n = 49		n = 48	
Speed (s)	3.12 ± 0.48	3.22 ± 0.43	3.07 ± 0.40	3.20 ± 0.56
	n = 49		n = 48	
Balance (score *)	3.40 ± 1.01	3.68 ± 0.98	3.32 ± 0.95	3.24 ± 0.85
	n = 51		n = 50	
Strength (kg)	7.64 ± 2.06	7.77 ± 2.12	7.74 ± 2.58	7.53 ± 2.61

* range: 1–5 (1 = better performance).

**Table 3:** Speed—Fixed Effects Parameter Estimates.

				95% C-Interval				
Names	Effect	Estimate	SE	Lower	Upper	Df	t	*P*
(Intercept)	(Intercept)	3.13	0.12	2.88	3.37	4.72	25.36	<0.001
Intake	without–with	0.11	0.05	0.02	0.20	93.00	2.50	0.014
Placebo_Type	DP–NDP	0.06	0.07	−0.08	0.20	86.98	0.88	0.382
Sex	girls–boys	0.16	0.07	0.02	0.30	87.64	2.19	0.031
BMI	BMI	−0.01	0.02	−0.06	0.04	88.82	−0.35	0.730
Intake xPlacebo_Type	without–with x DP–NDP	−0.02	0.09	−0.20	0.16	93.00	−0.23	0.818
Intake x Sex	without–with x girls–boys	0.14	0.09	−0.04	0.31	93.00	1.53	0.128
Placebo_Type x Sex	DP–NDP x girls–boys	0.21	0.15	−0.07	0.49	87.85	1.45	0.150
Intake x Placebo_Type x Sex	without–with x DP–NDP x girls–boys	−0.10	0.18	−0.45	0.26	93.00	−0.53	0.598

Footnote: R^2^ marginal: 0.06; R^2^ conditional: 0.64; BMI: body mass index; Placebo type: deceptive/nondeceptive placebo; Intake: with/without placebo.

**Table 4:** Balance—Fixed Effects Parameter Estimates.

				95% C-Interval				
Names	Effect	Estimate	SE	Lower	Upper	Df	t	*P*
(Intercept)	(Intercept)	3.43	0.16	3.13	3.74	5.48	21.83	<0.001
Intake	without–with	0.08	0.09	−0.09	0.26	93.00	0.95	0.343
Placebo_Type	DP–NDP	0.27	0.16	−0.04	0.57	88.02	1.72	0.088
Sex	girls–boys	−0.23	0.16	−0.54	0.08	89.54	−1.48	0.142
BMI	BMI	0.11	0.05	0.01	0.21	91.5	2.07	0.042
Intake x Placebo_Type	without–with x DP–NDP	0.36	0.18	0.02	0.71	93.00	2.07	0.041
Intake x Sex	without–with x girls–boys	0.26	0.18	−0.09	0.60	93.00	1.47	0.146
Placebo_Type x Sex	DP–NDP x girls–boys	−0.47	0.32	−1.09	0.15	90.07	−1.48	0.143
Intake x Placebo_Type x Sex	without–with x DP–NDP x girls–boys	0.05	0.35	−0.64	0.74	93.00	0.14	0.892

Footnote: R^2^ marginal: 0.09; R^2^ conditional: 0.61; BMI: body mass index; Placebo type: deceptive/nondeceptive placebo; Intake: with/without placebo.

## Data Availability

Data are available from the corresponding author.

## Supplementary Materials

The following supporting information can be downloaded at https://www.mdpi.com/article/10.3390/ejihpe14080161/s1, Table S1. Strength—Fixed Effects Parameter Estimates; Supplementary Material S1. Information sheet.
