# Supplementary material for: Placebo Effects on the Enjoyment of Physical Activity and Performance among Kindergarten Children: A Randomized Controlled Trial
Source: Eur J Investig Health Psychol Educ. 2024 Aug 22;14(8):2435–44. doi: 10.3390/ejihpe14080161 (PMC11353461; doi:10.3390/ejihpe14080161)
Supplement: Supplementary file 1 [file ejihpe-14-00161-s001.zip › ejihpe-3137838-supplementary.pdf]

Information Sheet

Study subject: The effect of placebos/OLPS on motivation and enjoyment of physical activity in kindergarten children

Dear parents,

A lack of physical activity can have negative health consequences for body and mind. This not only affects us as adults, but also our children. Promoting physical activity and making it attractive for them is therefore an important task in this context, which we want to address as part of a physical activity project at the University of Graz (Institute of Psychology).
In the study conducted by the Department of Clinical Psychology (headed by Prof. Dr Anne Schienle), the influence of placebos and open-label placebos (OLPs) on the motivation to engage in physical activity will be investigated in children between the ages of 3 and 6.

A placebo is a substance without a pharmacological active ingredient. The person receiving the placebo is led to believe that it is an effective intervention (e.g. medication). An OLP refers to a placebo where it is openly communicated that it is a placebo. Both placebos and OLPs have already been shown to be effective in research.

1. What is the purpose of the study?

The study (which was approved by the ethics committee of the University of Graz) aims to investigate whether the joy and motivation of kindergarten children to engage in physical activity can be increased by taking a placebo/OLP.
The attitude or expectation towards the administered substance plays a relevant role here. Our hypothesis: If you believe that the substance you take, which in reality contains no active ingredient, will make you stronger or make you run faster, then such an increase in performance can actually be induced. In this context, we want to investigate possible changes in the children's balance, grip strength and speed.

2. How is the study conducted?

The study will take place on a morning/afternoon on the premises of the kindergarten (time required per child: approx. 10-15 minutes). Three movement stations will be set up to assess balance skills using a balance board, grip strength using a hand dynamometer and speed by doing a short sprint. Each child completes the exercise tasks two times.
In one of the two rounds – before completing the exercises – each child is given a placebo or OLP in the form of a glass bottle with a spray head on it. This contains conventional tap water, which is sprayed onto the tongue in 3 pump doses. If the child receives a placebo, it is told that he/she is receiving a "magic potion" that will make him/her faster, stronger and better at balancing. If the child receives an OLP, it is openly told that there is normal water in the bottle. However, he/she is encouraged to "imagine" that taking the water will improve his/her own strength, speed and balance. Shortly before taking the placebo/OLP and after completing the exercise tasks, each child is asked how much he/she

believes that taking the water will help or has helped with task performance. After both rounds, all participants are asked how much fun they had with completing the exercises.

3. Are there any risks, complaints and side effects?

There are no health risks associated with this study. The placebo/OLP is normal tap water.

4. What data is collected during the study?

The following personal data is collected: name, gender, age, weight, height, emotional state at the time of the test, enjoyment of the test, subjectively perceived effectiveness of the placebo/OLP.

5. How is the protection of personal data guaranteed?

To ensure the protection of personal data, a subject identification code is generated, which is used for further processing or evaluation steps of the data.

6. How/by whom will the data collected during the study be used?

Only the study leader and her staff have access to the confidential data in which you or your child are mentioned by name. The data will only be passed on for statistical purposes. Neither you nor your child will be mentioned by name. You and your child will also not be named in any publications of the data from this clinical study.

7. Are there any costs for the participants?

Participation in the study does not lead to any costs for you or your child as a study participant.

8. Opportunity to discuss further questions

If you have any further questions in connection with this study, please do not hesitate to contact the study leader or the following contact persons. At the end of the study you will receive a written summary of the study results.

- Univ.-Prof. Dr. Anne Schienle: anne.schienle@uni-graz.at
- Marlies Stopper, MSc: marlies.stopper@uni-graz.at

9. Purpose and legal basis of the data processing

We (Clinical Psychology, University of Graz; Prof. Dr. Anne Schienle) - would like to further process and evaluate the data collected during the study on the basis of the legitimate and public interest of the University of Graz to promote and conduct research for scientific research purposes in the field of psychological research (see Art 6 Abs 4 DS-GVO iVm Art 6 Abs 1 lit e and f DS-GVO) and subsequently publish the evaluation results in aggregated and completely anonymized form.

Storage period: The raw data of the feedback, which form the basis for the aggregated and anonymized evaluation results of the publication, remain stored in the institution for ten years with access protection for the purpose of ensuring good scientific practice.

Transmission: Your personal data and that of your child will be evaluated and anonymized exclusively within the university in connection with the study. These aggregated and anonymized evaluation results will subsequently be published.
Rights: In connection with the processing of your personal data as well as that of your child, you have the so-called "data subject rights" at all times (such as the right to information pursuant to Art. 15 GDPR; the right to rectification pursuant to Art. 16 GDPR; erasure pursuant to Art. 17 GDPR or to restriction of processing pursuant to Art. 18 GDPR).With regard to the specific processing, the right to object to the processing of the collected data is particularly relevant, if this is justified for reasons arising from your particular situation (see Art 21 GDPR).

If you wish to lodge a justified objection to this further processing, as well as for the possible assertion of other data subject rights, please send an email to: anne.schienle@uni-graz.at.
In addition, you have the right to lodge a complaint (Art 77 GDPR) with a supervisory authority, in Austria this is the Austrian Data Protection Authority, Barichgasse 40-42, 1030 Vienna, telephone: +43 1 52 152-0, e-mail: dsb@dsb.gv.at.

Contact:

Universität Graz, Institut für Psychologie, 8010 Graz, Mail: anne.schienle@uni-graz.at

You can reach our data protection officer at: dsba@uni-graz.at

Please address general data protection enquiries to: datenschutz@uni-graz.at

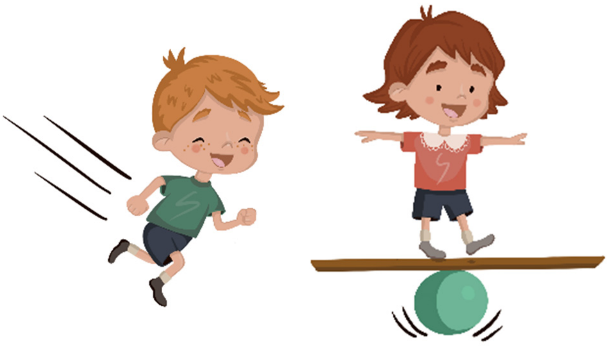

Consent Form

Please only sign the consent form

- if you have fully understood the nature and procedure of study,
- if you are willing to consent to your child's participation and
- if you are aware of your rights and those of your child as a participant in this study.

✓ I have read and fully understood the information about this study.

- ✓ I am aware that participation is voluntary and that consent to participate can be withdrawn at any time without any disadvantages.
- ✓ I agree that the data collected as part of this study may be obtained. The provisions of the Data Protection Act will be observed when handling the data.
- ✓ I can receive a copy of the declaration of consent on request. The original will remain with the study leader.

I ____ (surname, first name), born on _________________, resident in _____________________________, hereby give my consent for my child __________________________________ (child's name), born on ______________________, to participate in the above-mentioned study.

____
Signature

Table S1. Strength - Fixed Effects Parameter Estimates

				95% C-Interval				
**Names**	**Effect**	**Estimate**	**SE**	**Lower**	**Upper**	**df**	**t**	**p**
(Intercept)	(Intercept)	7.56	0.49	6.61	8.52	4.89	15.52	< .001
Intake	without - with	-0.02	0.12	-0.26	0.22	97.00	-0.14	0.886
Placebo_Type	DP - NDP	-0.08	0.41	-0.89	0.73	91.26	-0.20	0.845
Sex	girls - boys	-0.81	0.42	-1.63	0.02	92.67	-1.92	0.058
BMI	BMI	0.12	0.14	-0.15	0.39	94.02	0.85	0.398
Intake ✻ Placebo_Type	without - with ✻ DP - NDP	0.36	0.25	-0.13	0.84	97.00	1.45	0.150
Intake ✻ Sex	without - with ✻ girls - boys	-0.30	0.25	-0.79	0.18	97.00	-1.22	0.224
Placebo_Type ✻ Sex	DP - NDP ✻ girls - boys	0.56	0.84	-1.09	2.20	93.05	0.66	0.509
Intake ✻ Placebo_Type ✻ Sex	without - with ✻ DP - NDP ✻ girls - boys	-0.33	0.49	-1.30	0.64	97.00	-0.67	0.504
